# Implementation of Guidelines on Prevention of Coercion and Violence (PreVCo) in Psychiatry: Study Protocol of a Randomized Controlled Trial (RCT)

**DOI:** 10.3389/fpsyt.2020.579176

**Published:** 2020-09-15

**Authors:** Tilman Steinert, Andreas Bechdolf, Lieselotte Mahler, Rainer Muche, Johanna Baumgardt, Felix Bühling-Schindowski, Celline Cole, Marie Kampmann, Dorothea Sauter, Angelika Vandamme, Stefan Weinmann, Sophie Hirsch

**Affiliations:** ^1^ Center for Psychiatry Suedwuerttemberg, Ulm University, Ravensburg-Weissenau, Germany; ^2^ Department of Psychiatry, Psychotherapy and Psychosomatic Medicine, Vivantes Hospital Am Urban and Vivantes Hospital am Friedrichshain, Berlin, Germany; ^3^ ORYGEN, National Center of Excellence of Youth Mental Health, University of Melbourne, Melbourne, VIC, Australia; ^4^ Department for Psychiatry and Psychotherapy, University Hospital Cologne, Cologne, Germany; ^5^ Department of Psychiatry and Psychotherapy (CCM), Charité-University Medicine Berlin, Berlin, Germany; ^6^ Institute of Epidemiology and Medical Biometry, University of Ulm, Ulm, Germany; ^7^ Department of Psychiatry and Psychotherapy, Center for Psychosocial Medicine, University Medical Center Hamburg-Eppendorf, Hamburg, Germany; ^8^ Department of Psychiatry and Psychotherapy, University Psychiatric Hospital Basel (UPK), Basel, Switzerland

**Keywords:** psychiatry, coercive measures, restraint, seclusion, violence, clinical guidelines, evidence based treatment, quality management

## Abstract

**Background:**

Coercive measures are among the most controversial interventions in psychiatry. There is a large discrepancy between the sheer number of high-quality guidelines and the small number of scientifically accompanied initiatives to promote and evaluate their implementation into clinical routine. In Germany, an expert group developed guidelines to provide evidence- and consensus-based recommendations on how to deal with violence and coercion in psychiatry.

**Methods:**

The study presented examines whether coercive measures on psychiatric wards can be reduced by means of an operationalized implementation of the guidelines “Prevention of coercion: prevention and therapy of aggressive behavior in adults”. Out of a set of 12 interventions offered, wards are free to choose three interventions they want to implement. The primary outcome is the number of coercive measures per bed and month/year. Secondary outcomes are cumulative duration of coercive measures per bed and month/year. The most important control variable is the number of aggressive incidents. We plan to recruit 52 wards in Germany. Wards treating both voluntary and compulsorily admitted patients will be included. A 1:1 stratified randomized controlled trial will be conducted stratified by the amount of coercive measures and implemented aspects of the guidelines. In addition to the control group analysis, a waiting list design allows a pre-post analysis for all participating wards of the waiting list group. A parallel qualitative study will examine factors related to successful implementation and to successful reduction of coercion as well as relevant barriers.

**Discussion:**

We are planning a nationwide study on the implementation of evidence- and consensus-based guidelines in psychiatric hospitals. This study intends to promote the transfer of expert knowledge as well as results from clinical trials into clinical routine with the potential to change supply structures in mental health sector.

**Clinical Trial Registration:**

www.isrctn.com, identifier ISRCTN71467851.

## Introduction

Whether and under what circumstances the use of coercion in psychiatric treatment is justified is one of the oldest and most controversially discussed questions in psychiatry. Since psychiatric hospitals have long been assigned regulatory tasks in addition to medical ones, the discussion is caught between treatment and safety, patient rights and the rights of third parties. Today, most psychiatric and legal experts agree that coercive measures may only be used if other measures were not sufficient to avert danger from the patient or others. In 2011, 2016, and 2018, coercive measures in psychiatric care were the subject of landmark rulings by the German Federal Constitutional Court, each of which entailed significant changes to the Mental Health Laws (1–3).

The German Federal Constitutional Court has emphasized that mechanical restraint in particular should only be used as a “last resort” (2). In 2016, 6.7% of all admissions to psychiatric hospitals experienced freedom-restrictive coercive measures such as seclusion and restraint according to the data of the register in the German federal state Baden-Wuerttemberg (4). Relative to a population of approximately 11 million inhabitants, 30,386 of such measures took place (4). The subject has been classified as highly relevant by the German Association for Psychiatry, Psychotherapy and Psychosomatics (DGPPN) and is also the subject of numerous statements (5). Against this background, the Clinical Practice Guidelines “Prevention of coercion: prevention and therapy of aggressive behavior in adults” (6) was published in 2018. These are S3 guidelines, what means that they have achieved the highest methodological quality of guidelines developed in Germany and that they are evidence and consensus based taking into account all available scientific literature and the opinion of acknowledged specialists in the field. A comprehensive research of the scientific evidence as well as a structured consensus-finding process involving all relevant experts was incorporated into the recommendations. These guidelines define aggressive behavior as an interactive process. Thus, aggressive behavior in psychiatric institutions results from the interaction of individual experience and behavior on the part of patients, relatives and employees, situational characteristics and an escalation component. This means that aggressive escalations as well as coercive measures by psychiatric institutions can be modified. There is already a wealth of research on the reduction of coercion and violence (7). Measures which were effective in clinical trials were staff educational programs and regular training of the employees (8), enrichment of the ward environment, structured risk assessment and early interventions [e.g., Brøset Violence Checklist (9)], individualized treatment planning, especially advanced care planning (10) for patients who already experienced violence and coercion, as well as debriefing techniques. Interventions were especially helpful if they were combined with each other, incorporated organizational changes and were endorsed by the management of the clinic. Therefore, several complex interventions consisting of different measures were developed in the past few years, e.g., internationally Six Core Strategies (11) and Safewards (12, 13) and in Germany Weddinger Modell (14). While the Six Core Strategies (11) include top-down-elements focusing on the organization of a psychiatric ward or clinic, Safewards emphasizes the communication among patients and staff on a specific ward (12). The Weddinger Modell, developed in 2010 in Berlin, Germany, is an innovative model of psychiatric care focusing on recovery, participation, supported decision-making and the prevention of coercive measures on psychiatric wards (14).

Until recently, high-quality guidelines were mainly available for acute emergency situations, as, e.g., for aggressive agitation (15). The German clinical practice guidelines, on the other hand, also attempt to summarize the preliminary work on de-escalation and violence prevention in practical recommendations. They were developed with the substantial participation of patient and family associations and offer detailed evidence- and consensus-based recommendations for the first time on how psychiatric clinics can meet these challenges.

Dealing with violence and coercion is regarded as one of the most important aspects of the quality of psychiatric care (16). For many years, the topic has also been classified as highly relevant in Germany, especially against the background of constantly increasing demands for respect of patient autonomy on the one hand and the protection of employees’ rights by the Occupational Health and Safety Act on the other hand. In addition, patient and family associations are calling for improvements particularly in this area. People with mental health problems and their relatives perceive seclusion and restraint as anti-therapeutic. In a qualitative study, measures restricting freedom were regarded as human rights violations even if they were recognized by affected patients as necessary to avert risks (17). The Federal Constitutional Court has clarified that there is a considerable need for action with regards to reducing the use of restraints. The named guidelines show how this can be achieved, taking into account the available evidence. In 2018, the Gemeinsamer Bundesausschuss (Federal Joint Committee, a committee that determines what is paid for by the statutory health insurance fund) even addressed this issue in relation to the structure of the remuneration system in the German health system.

Worldwide, it is found to be highly problematic that there is considerable variability in the frequency of application of coercive measures between hospitals (18, 19), which could also be confirmed for Germany (20, 21). Therefore, beginning in the years after 2000, a common system of data registration and hospital comparisons had been introduced. This was accompanied by continuous efforts of a quality circle, showing that this variance can be reduced (22). This also suggests that implementation of measures to reduce coercion could have the potential to reduce coercive measures and the variance between clinics. In this line, we used the guidelines published in 2018 to derive operationalized recommendations for a) care regions, b) psychiatric hospitals, and c) psychiatric wards. We deemed it important to provide only recommendations that are measurable in terms of the degree of implementation. The recommendations for implementation were adopted in November 2018 by the German Association for Psychiatry, Psychotherapy and Psychosomatics (DGPPN).

The recommendations can be implemented as a complex intervention. Thus, they arrive directly at the level of the psychiatric ward, measurable with implementation indicators and at the level of patient care in terms of impact on coercive measures and assaults. A sustainable goal of the project is expected to be the improvement of care processes in one of the most sensitive areas of psychiatric treatment.

Due to their very heterogeneous size and structure, entire hospitals are difficult to address and to evaluate. This applies even more for the care regions, since a large number of other actors such as police, courts, and service providers are involved. As with most international evaluations of such interventions, the most appropriate target level is the ward level. To date, there is a remarkable disparity between the high number of evidence-based guidelines and the comparatively low number of concrete implementation recommendations and the even lower number of implementation studies and evaluations. While the implementation of peri-operative interventions, for example, seems comparatively easy to operationalize and measure, the challenges regarding the diverse and complex recommendations in psychiatric care are obviously greater. A review in 2017 identified only 17 studies comparing guideline implementation strategies versus routine care and found no consistent effects on provider performance (23). Some positive results can be mentioned: In the Netherlands, it was recently demonstrated that guidelines on suicide prevention could be successfully implemented in 24 institutions. However, there was no evidence at the level of patient-related outcomes (24). In Canada, an implementation strategy for schizophrenia guidelines was developed and successfully implemented at a single hospital (25). The high costs involved in developing high-quality guidelines and the low benefits of not applying them have been the subject of repeated criticism. Concerns have even been voiced by guideline authors themselves in recent years (26).

The aim of this study is to accomplish the final and most difficult step of guideline implementation: The first step was the development of guidelines together with many experts and all relevant organizations of professionals, patients, and relatives. The second step was the development of the 12 concrete recommendations for implementation and instruments to measure the effects at the level of psychiatric wards. There is good evidence for all 12 individual elements and a high consensus among 23 expert groups, including professionals, patients, and their relatives who were involved in the development of the guidelines. The third step was a pilot study in five psychiatric hospitals ending in December 2019, testing the feasibility of the procedure and the interventions (27). The wards were accompanied for six months by psychiatrists and nursing consultants from the study team. It was agreed in advance that three of the twelve recommendations would be newly implemented. Wards were only included in the pilot study if the nursing and medical directors agreed to support the implementation. At the beginning of the six months implementation period, a one-day awareness workshop with the medical and nursing ward management, the team members, and the next higher level of medical and nursing hierarchy was held. Consultants and ward staff commonly evaluated (i) which recommendations were already realized in the ward and to which extent and (ii) which recommendations should be introduced as part of the pilot study. Given the urgency of the topic, it can be assumed that so far there is no psychiatric hospital which has until now not implemented any interventions or strategies to reduce coercion. At the same time, no clinic has fully implemented all recommendations suggested in the guidelines. The consultants and the teams agreed upon a timetable with milestones. Two half-day intermediate workshops for monitoring and further counseling were scheduled. In the meantime, the wards always had the opportunity to contact their consultants by telephone or e-mail.

The fourth step will be a randomized controlled study with sufficient statistical power to test the effectiveness of the intervention on a ward-basis. For this step the design and methods will be described in this study protocol.

## Methods

### Study Design

This multicentre study applies a mixed-methods design. In this interventional trial, wards are randomized in a 1:1 ratio to either an intervention or a control condition (waiting list), stratified by the amount of coercive measures per bed and month/year and the implemented aspects of the guidelines to matched pairs. In addition, a waiting-list control design allows for a pre-post analysis for participating wards of the waiting list group. Furthermore, this design allows for analyzing if observer effects already lead to a reduction of coercive measures between the baseline and the start of the intervention as well as for assessing potential spill-over effects in the control group during the waiting time. After 12 months, control wards will receive the intervention. Pre-post analysis will also be done or interventions wards which provide additional data of one year of follow-up after the intervention. Moreover, a complementary qualitative trial in form of interviews with participating staff will be performed.

### Participating Wards

The study will involve 52 psychiatric wards in 52 hospitals in Germany treating patients with severe mental illness, including involuntary treatment. Prerequisites for participation are a written declaration of support by the hospital management and the ward management, the willingness to work with agreements on objectives, and the provision of the cumulative evaluation of the outcomes for research purposes. Wards of forensic psychiatry, child and adolescent psychiatry, and wards where mainly people suffering from dementia are treated are excluded from the trial.

### Recruitment of Wards

Two study centers are planned: One in north-eastern (Berlin) and one in the south-western (Ravensburg) of Germany. Each study center has to recruit and supervise 50% of the wards. Hospitals can participate with more than one ward. A contract will be signed with description of duties of each party. There are no financial incentives for participating hospitals, but continuous support and counseling by a research worker.

### Interventions

**Figure 1:**
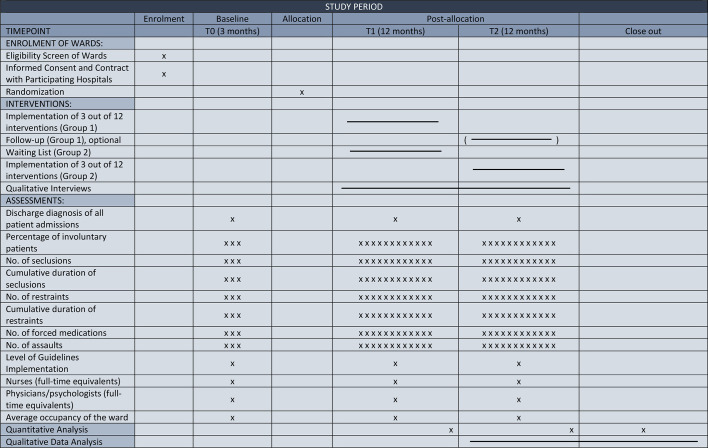
SPIRIT flow chart: Timeline and study procedures.

In the beginning, a first workshop to assess the present state of clinical practice together with the ward team will be held. This data will be used for matching and randomization of the wards. The possible interventions will be presented to the ward team within a second awareness workshop. The research assistant will support staff to select appropriate interventions. Subsequently, the research assistant will provide advice on how to realize the intervention and will establish action plans with targets and timelines in close collaboration with the ward team. During the implementation process, an interactive counseling will be realized with selected key persons on the ward *via* telephone and email. Key persons will also receive advice on collecting and delivering data. A third workshop will be during the intervention period. At the end of the study period, a fourth visit of the team will be realized by the research worker. The planned timeline is shown in **Figure 1**. The 12 offered and recommended guideline-based interventions are as follows:

1. Implement a standardized recording of coercive measures and aggressive incidents with the possibility of regular evaluation at ward level.
2. Implement internal standards adapted to the guidelines regarding the indication, initiation, review, documentation, and debriefing of coercive measures, or review existing standards, as appropriate.
3. Hold a monthly team meeting, chaired by the department or ward manager, to analyze data on coercive measures and aggressive incidents and discuss the background.
4. Implement a training plan for all employees with patient contact in de-escalation/aggression management and ensure that all employees receive training at least once every two years.
5. Ensure that any coercive measures restricting or depriving freedom (restraint, seclusion) are accompanied by continuous observation and personal care.
6. Ensure that debriefings after coercive measures with the affected patients take place and are documented.
7. Employ or involve peers on the ward.
8. Create an action plan for the aggression-reducing design of the spatial environment on the ward and review it annually.
9. Introduce a risk assessment with the Brøset Violence Checklist (BVC) or another instrument for all patients at risk according to clinical assessment and make sure that clinical consequences result. For scores above BVC 2, e.g., the patient is contacted for de-escalation within half an hour, usually by at least two persons.
10. During debriefing after a coercive measure, recommend all patients to draw up a joint crisis plan for the prevention of future coercion.
11. Introduce measures to ensure guideline-based pharmacotherapy [based on the guideline with regard to aggressive behavior, but also the disorder-specific other guidelines (“guideline-based treatment of the underlying disease”)], and, e.g., monthly random check or hold regular meetings during rounds
12. Introduce complex interventions for reducing coercion that can be operationalized into individual modules (e.g., Safewards, Weddinger Model, Six Core Strategies).

Due to the heterogeneous initial situation of each ward need for action will have to be determined for each ward individually. To this end, research workers and ward managers will jointly evaluate the status of implementation at the first visit. Afterwards, ward staff will discuss and declare their preferred interventions. A set of up to three selected interventions will be the ward’s working focus during the interventions period. However, e.g., implementing a complex intervention such as Safewards is considered as sufficiently demanding to be selected alone. The underlying idea is that it would be unrealistic to fully implement all 12 recommendations within 12 months. Demand this would probably result in a low fidelity to the intervention. In addition, we assume that it makes little sense to require the implementation of elements of the intervention that might be viewed as unfeasible or undesirable by the staff. On the other hand, this means that the intervention will not be fully completed after the study period and cannot have unfolded its full power. Rather, implementation has to be considered as a process over several years, with the study intervention only boosting an initial period. The selection of individually fitting interventions by participating wards means that not all participants will deliver identical interventions. The qualitative interviews are intended to explore, among other things, why interventions are selected and which barriers had arisen during implementation.

### Standard of Care on Control Wards

The legal requirements (mental health laws, civil code) already require the use of coercion to be limited to a necessary minimum. The participating wards should continue to apply their current strategies to prevent coercion and violence, even if they are randomized into the control group and even if these strategies are also mentioned in the 12 interventions. New structured programs especially with external supervision to reduce coercion and violence further should not be implemented in this phase. However, control wards will be informed about the program and its possibilities and spill-over effects cannot be ruled out. To detect such effects, a second measurement of implementation status and outcomes will take place on the control wards before the beginning of the intervention after the waiting list status.

### Outcomes

The primary endpoint will be the number of coercive measures per bed and month/year for each ward (intervention wards vs. waiting list wards). This dependent variable includes forced medication, physical and mechanical restraint as well as seclusion. We chose this primary outcome according to its frequent use in the international literature in studies on ward level: 61 out of 78 studies on the topic reduction of coercive measure chose this endpoint (7). By the calculation per bed ward of different sizes become comparable. We are using aggregated information derived from routine data of the wards. No personal data is collected.

Secondary outcome will be cumulative duration of freedom-restrictive measures (seclusion, restraint) per bed and year (in hours) as well as the change before and after the intervention for each ward of the waiting list group (waiting list period vs. intervention period).

Important variables to be controlled for will be

status of implementation at the first visit (measured with a Likert scale, see next par).
number of aggressive incidents per bed and year (measurement see next par).
staffing levels
percentage of involuntary admissions

### Instruments

At the beginning of the trial, a joint evaluation by study staff and the ward team will be carried out for each of the 12 recommendations on a scale from 0 (= not implemented at all) to 9 (= fully implemented). This weighting is realized by with a Likert scale with typical anchor examples for each recommendation for the scores 0, 3, 6, and 9.

For the recording of violent incidents, the guidelines recommend the use of the SOAS-R (28). This instrument is most widespread throughout Europe and has already been introduced at many clinics in Germany. It is a well validated instrument with a score ranging from 0 to 22 (higher ratings stand for more severe incidents). In order to compensate problems of under- and over-reporting as much as possible, events will be considered only above a minimum score of 10 points. This allows both individual events and their severity to be taken into account. Furthermore, SOAS-R outcomes can be treated as continuous variables also. However, some of the participating hospitals will have introduced other, possibly less standardized instruments in their clinical practice. To avoid unwanted effects of increasing awareness in the course of the introduction of a new instrument, these hospitals will be advised to continue with their introduced instrument without any change in the practice of application.

### Sample Size

The primary outcome (number of coercive measures per bed and year) will be compared in a matched pair design, using a special stratification for matching of wards. Since the magnitude of the effect is not known for formal sample size estimation, the case count estimation is based on a realistic estimation of feasibility. The realistic number of wards participating in a nationwide study is assumed to be about 50. Using a paired two-sided t-test, using the primary outcome as continuous variable and the significance level of 5% and a power > 80%, an effect size of 0.6 can be detected with this case number (nQuery 8.1 Professional, exactly: 24 pairs = 48 wards). From a clinical point of view, an even larger effect is expected from the implementation of clinical practice guidelines. Since data is probably not normally distributed, however, the Wilcoxon test for paired data should be used as an evaluation method. This requires about 5%–10% more observations (or rather pairs) to compensate for the loss of power. Accordingly, a case number of 26 pairs (=52 wards) was specified. Drop-outs are not to be assumed in this trial, since the relevant outcome data must be collected from each ward for purposes of routine reporting anyway.

### Randomization

The wards will be randomized to intervention group or waiting-list control group by a 1:1 randomization. The participating wards will be matched in pairs following the best-fit principle according to the two criteria baseline frequency of coercive measures and initial value of the Likert scale for conformity with the implementation goals. Because this is a crucial confounder, the randomization will be done stratified accordingly. To get a matched-pair design to control at most for this confounder, the wards will be ranked by this variable (see “instruments”). In each of pairs in this ranked list a block randomization with a block size of two will be done by the randomization-software ROM in the independent Institute of Epidemiology and Med. Biometry, Ulm University. The matched wards must not belong to the same hospital in order to avoid spill-over effects.

### Data Collection

The documentation and reporting of coercive measures is legally stipulated in the federal State of Baden-Württemberg in a standardized form with a manual containing definitions of different coercive measures in the mental health law. All hospitals must record raw data in this regard and transmit it to the federal state-wide register. Standardized forms for electronic data entry are already available and there is a well-established practice in this regard. The possibility of an evaluation for individual clinical units and time periods is therefore possible without large additional expenditure. The raw data stored in the electronic medical records will be used. Data will be fully anonymized and it will no longer be possible to assign information to individual patients, even for clinical staff. The possibility of cumulative, care unit-related evaluation is a prerequisite for participation in the study. The raw data is stored in the respective clinics together with the corresponding medical records for the legally required retention period. For the study purpose, no raw data is transferred to the evaluation center, but only cumulative evaluations according to the described outcomes. Participating hospitals will be provided with tables to fill in for this purpose. Because no patient-related data is collected, no anonymization or pseudonymization is required. The cumulative data is stored at the evaluation site in connection with the relevant ward. A special concept for the purposes of data protection was established according to the requirements of the funding agency.

### Statistical Analysis

The main outcome number of coercive measures per bed and year is evaluated by comparing the ward pairs by the paired Wilcoxon test at the significance level of 5%. Secondary outcome is also evaluated by the paired Wilcoxon test. In order to control confounding and reduce bias, the analyses are supplemented by multiple regression models (linear mixed models and/or generalized conditional logistic regression models) including possible covariates (number of admissions, average occupancy, proportion of involuntarily treated patients, average staffing levels). These analyses will be interpreted explorative. Raters will not be blinded due to the fact that control and interventions wards will be analyzed at different time points and additional analysis (pre-post comparison) are needed for the control wards.

### Qualitative Analysis

For the qualitative evaluation, researchers will interview one or two selected key persons at each of the participating wards during the course of the implementation. It is planned that one researcher at each study center will be responsible for the implementation and for identifying the key persons. The key persons will be interviewed *via* telephone by another researcher. An interview guide will be prepared for this purpose. The interviews will be recorded, transcribed and subjected to a qualitative content analysis according to Mayring (29) using the MAXQDA program. Different categories will be developed to identify typical barriers and favorable factors for a successful and sustainable implementation. This content analysis technique is broadly applied in social sciences to evaluate large quantities of material from semi-structured interviews. It allows to build categories of content and to count certain text components (e.g., aspects of stigmatization, safety feeling and ward atmosphere).

## Discussion

The outlined trial will be a nationwide mixed-method study on the implementation of evidence- and consensus-based guidelines on reduction of coercion in mental health care. To our knowledge, there were only smaller studies on guideline implementation in psychiatry in general with inconsistent results so far (30). With regard to a related topic of the objective addressed here, the process of involuntary hospital admission in the USA was improved by implementing guidelines (31). In Germany, complex guidelines on handling psychotropic medication during agitation could be implemented in a cluster-randomized design in nine nursing homes in Berlin and were compared with nine control facilities; significant improvements on the level of patient-related outcomes were observed (32). In 15 registered psychiatrist offices in Munich, a computer-assisted decision aid for therapy decisions based on schizophrenia guidelines was implemented, which at least initially showed improvements over a control group (33). Another relatively small study in Germany referred to the implementation of the medication in schizophrenia guidelines on four wards, with relative improvements in a pre-post design (34).

With the potential to promote relevant changes in mental health care, this study is intended to realize the transfer of expert knowledge as well as the results from clinical trials into clinical routine. The main risks for successful guideline implementation lie in the current scarcity of human resources in hospitals. The study only makes experts or consultants available to the wards, but not additional nursing or therapeutic personnel. It is well-known that the implementation of measures to reduce violence and coercion can be time-consuming and personnel-intensive. Additionally, large studies harbour the risk that implementation varies considerably in quality and extent across participating centres, resulting in the worst case scenario of zero effects on average, as it happened in a study on joint crisis plans (35, 36). This risk is to be countered by recording the score of the Likert scale developed especially for this purpose.

## Ethics Statement

The studies involving human participants were reviewed and approved by The project was approved by the Ethics Committee of the University of Ulm on September 4th, 2019, No. 55/19. Written informed consent for participation was not required for this study in accordance with the national legislation and the institutional requirements.

## Author Contributions

TS and RM designed the study. TS, SH, and RM were involved in preparation and procedure. SH wrote the first draft. TS contributed to the draft. RM, AB, LM, JB, FB-S, CC, MK, DS, AV, and SW contributed to the final manuscript. All authors contributed to the article and approved the submitted version. TS supervised the project.

## Funding

The study is funded by the Innovationsausschuss beim Gemeinsamen Bundesausschuss (project no. 01VSF19037). The funders had no role in study design or data collection.

## Conflict of Interest

The authors declare that the research was conducted in the absence of any commercial or financial relationships that could be construed as a potential conflict of interest.
